# Biological reconstruction in the treatment of extremity sarcoma in femur, tibia, and humerus

**DOI:** 10.1097/MD.0000000000020715

**Published:** 2020-07-02

**Authors:** Weitao Yao, Qiqing Cai, Jiaqiang Wang, Peng Zhang, Xin Wang, Xinhui Du, Xiaohui Niu

**Affiliations:** a Bone and Soft Department, the Affiliated Cancer Hospital of Zheng Zhou University, He Nan cancer Hospital; b Department of Orthopedic Oncology Surgery, Beijing Ji Shui Tan Hospital, Peking University. Beijing, China, No. 31 Xin Jie Kou Dong Jie, Xi Cheng District, Beijing, China.

**Keywords:** biological reconstruction, complication, extracorporeal devitalization and reimplantation, malignant bone tumor, survival rate

## Abstract

To understand the feasibility, clinical effect, and complications related to biological reconstruction techniques for long limb malignant bone tumors after excision.

This retrospective study included eighty patients with malignant bone tumors treated at our hospital between January 2007 and January 2019. After tumor resection, 52 cases of intercalary and 28 cases of osteoarticular bone grafts were used. The implanted bone included devitalized recycling bone, fibular, and allograft.

The average follow up period was 42.19 months for 80 patients, among whom 15 (18.75%) died. The 5-year EFS and OS were 58% and 69%, respectively. The average length of the replanted bone was 18.57 cm. The MSTS scores of intercalary and osteoarticular bone grafts were 87.24% and 64.00%, respectively. In 23 cases (44.23%) of metaphyseal and 26 cases (32.5%) of the diaphysis, bone graft union was obtained at the first stage. The factors affecting bone union were the patient's gender, age, devitalization bone methods and whether the implanted bone was completely fixed. Postoperative complications included delayed bone union in 15 patients, fractures in 25 cases, nonunion in 22 cases, bone resorption in 14 cases, and postoperative infection in 4 cases. Twenty-eight cases of bone grafting required revision surgery, including replacement of internal fixation, autologous bone graft, debridement, removal of internal fixation, and replacement with prosthetic replacement.

Biological reconstructions with massive bone grafts are useful in the reconstruction of certain malignant extremity bone tumors after wide excision.

## 1 Introduction

Bone and soft tissue sarcomas, which represent 1% to 10% of all malignant tumors, are mainly found in growing or adult patients.^[1]^ Over the past couple of decades, therapy of these rare sarcomas has considerably changed because of the several successful interdisciplinary treatment strategies.^[2,3]^ A dramatic increase in the survival rates that went from 20% to 60% to 80% was achieved by the introduction and improvement of adjuvant chemo- and radiotherapy in bone and soft tissue sarcomas.^[4,5]^

The bone and soft tissue sarcomas most frequently originate in extremities.^[6,7]^ Because they tend to be very invasive, they may extend into the boundary compartments and invade the neurovascular bundle. Nowadays, the tumor can be eradicated either by amputation or limb-salvage surgery.^[7,8]^ Limb salvage surgery, which is the first choice of treatment by both patients and surgeons, is primarily performed to adequately excise tumors while preserving the particular limb. It consists of complete removal of a malignant tumor with wide margins and reconstruction of the limb with an acceptable oncologic, functional, and cosmetic result. The reconstruction methods after massive tumor bone resection are mainly based on tumor prognosis, the remaining bone and soft tissue, and patient/family expectations.^[9,10]^

Endoprosthetic reconstruction is widely used because of its convenience, immediate weight-bearing, and good functional outcomes.^[11]^ Nevertheless, this approach also has certain shortcomings, such as high financial cost and complications, including infection, loosening, wear, and breakage in the long-term of follow up.^[12,13]^ Reconstruction with a prosthetic replacement is not suitable for children who have not yet reached skeletal maturity.^[8]^ These are inevitable difficulties associated with prosthetic replacement and are the reason why many orthopedic surgeons strive for long-term results employing biological reconstruction methods.

The biological reconstruction implies that the diseased bone is replaced by an expendable, which completely replaces the role of the removed portion and can be classified as either viable or nonviable. The former involves vascularized bone graft and lengthening of the bone,^[14]^ while the latter involves devitalized tumor bone^[15]^ and allograft. Vascularized bone grafting has been reported as a safe and reliable procedure for reconstruction of segmental skeletal defects resulting from intercalary resection of malignant bone tumors. It can result in the immediate recovery of physiological blood supply to the cellular elements of the graft, thus maintaining its viability and mechanical stability at the recipient-graft junction. The limitations include the scarce supply of donors, inadequate adaptation for the recipient, late stress fracture, and the high technical demands of the operation.^[16,17]^ Distraction osteogenesis with bone transport techniques for such significant bony defects involves a lengthy period in a cumbersome frame and a high incidence of complications, thus making it far from an ideal solution for these challenging cases.^[14]^ Massive allografts are widely used because of biological fixation and attachments for soft tissue anchorage, with the problems of procurement, storage, immunological responses, and possible infection. Over recent years, there has been a great interest in recycling the tumor bone itself by various methods of sterilization and re-implantation. Extracorporeal devitalization and re-implantation of the resected bone, which can be achieved by heating, irradiation, or freezing thanks to its biological nature, is an excellent alternative method being much more economical and durable.^[15,18,19]^ Biological reconstruction by recycling of resected tumor-bearing bone is prevalent in certain Asian countries for socio-religious reasons.

The aim of this study was to examine the effects of biological reconstructions, including massive allografts, autologous fibula, and the recycle tumor bone in the replacement of long bone, such as femur, tibia, and humerus in extremity sarcoma patients.

## 2 Materials and methods

### 2.1 Case selection

A total of 1032 patients with a malignant bone tumor of extremities who were diagnosed and treated at our hospital between January 2007 and January 2019 were initially identified and selected from medical records. Further screening was performed according to the location of the disease (including femur, tibia, and humerus) and treatment methods. A total of 157 patients underwent biological reconstruction of the defects after malignant bone and soft tissue resection. Through clinical and image data collection, and by excluding the patients who were lost to follow up (42 patients), repeated registration (12 patients) and were postoperatively followed-up for less than six months (23 patients), a total of 80 patients with complete data were finally included.

Ethical approval was obtained from the Medical Ethics Committee of the Henan Cancer Hospital (No. 182102410001).

### 2.2 General information

Among 80 patients, 48 were male, 32 were female, with a male/female ratio of 1.5. The age of the patients ranged from 8 to 64 years, with an average age of 19.62 years. The leading age group was 10 to 20 years old, with a total of 47 cases (58.8%). The incidence sites included: femur in 42 cases, and tibia and humerus in 19 cases, respectively. There were 69 osteosarcomas (including 62 cases of conventional osteosarcoma, four cases of low-grade central osteosarcoma, two cases of parosteal osteosarcoma and one case of telangiectatic osteosarcoma), 5 Ewing sarcomas, 4 chondrosarcomas, and 2 malignant soft tissue sarcomas confirmed by pathology (see Table 1).

**Table 1:**
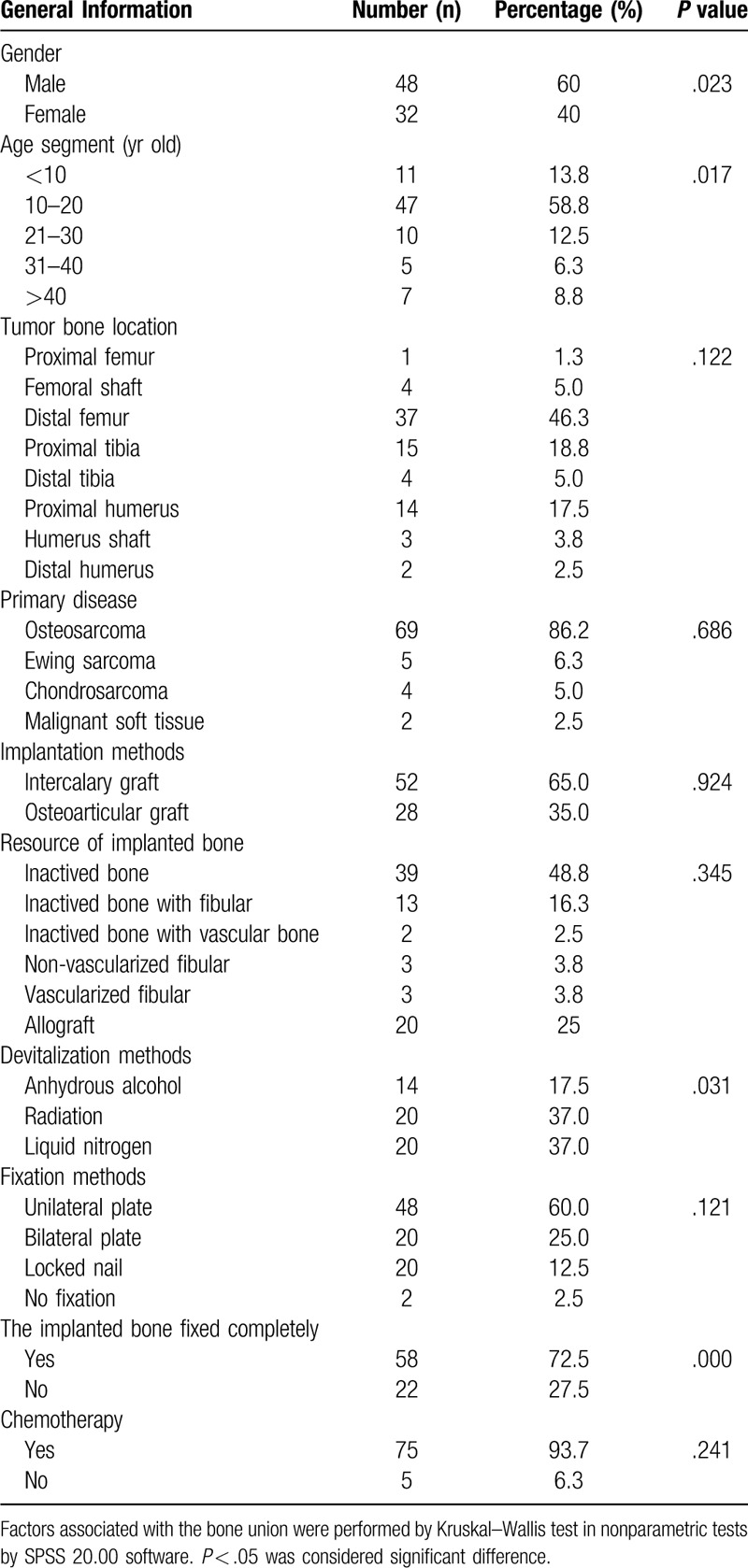
Baseline characters of 80 patients and the factors of implanted bone healing in 1 stage.

### 2.3 Examination and therapy before operation

The initial assessment of these patients was carried out according to the current protocol of our institution, which includes plain X-ray, MRI, CT scan, bone scan, CT scan of the lungs, and standard laboratory investigations. Following the imaging survey, all patients underwent a carefully planned core needle or open biopsy and histopathological examination. Following the biopsy, the tumors were staged according to the modified system of the American Joint Committee on Cancer.^[20]^ There were 4 cases in Stage IA, 2 cases in Stage IB, 4 cases in Stage IIA, 65 cases in Stage IIB, and 5 cases in Stage IIIA.

In neo-adjuvant therapy, 2 standard cycles of chemotherapy were taken in the 74 osteosarcomas and Ewing patients, and radiotherapy of 30 Gy was given to the two soft tissue sarcoma patients. The efficacy of chemotherapy and the exclusion of metastatic lesions were evaluated by imaging data, after which the surgical treatment was performed. No other therapy was given to the remaining four chondrosarcoma patients.

### 2.4 Operation procedure

The first step included a wide en bloc excision, including all involved compartments as planned through the imaging study. Intercalary resection was done in 52 cases, and osteoarticular resection in the remaining 28 cases.

The second step was the preparation of implanted bone, which included: ① debridement and devitalization of the resected bone; dissection of all the soft tissue components of the tumor, reaming of the medullary canal, and removal or remain the attached soft tissues. The debrided tissue was sent for histopathological examination. The excised segments were devitalized in anhydrous alcohol for 30 minutes in 14 cases (plates and screws fixed before devitalization), with radiation of 50 Gy^[21,22]^ in 20 cases, or nitrogen for 20 minutes in 20 cases. The segments frozen by nitrogen were thawed at room temperature for 15 minutes, thawed in distilled water for 10 minutes before use.^[19,23]^ ② Harvesting the tibia with or without a vascular pedicle from the ipsilateral low leg. ③ Rewarming the allograft bone at room temperature, rinsing with normal saline, and bone plasticity according to the bone defect.

**Figure 1:**
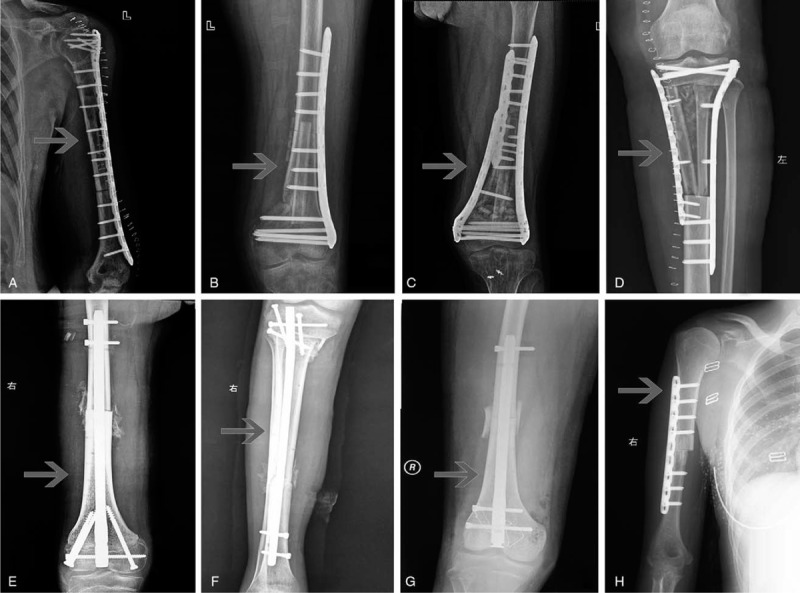
Different biological reconstruction methods (the arrows show the involved bone). A. Intercalary devitalized bone by anhydrous alcohol and fixed by a unilateral plate in an 18 years old female Ewing sarcoma patient at proximal humerus. B. Intercalary devitalized bone by radiation with fibular and fixed by a unilateral plate in a 15 years old male osteosarcoma patient at distal femur. C. Osteoarticular devitalized bone by liquid nitrogen with fibular and fixed by a bilateral plate in a 25 years old male osteosarcoma patient at distal femur. D. Intercalary fibular segments and fixed by a bilateral plate in a 23 years old female osteosarcoma patient at the proximal tibia. E. Intercalary allograft replacement fixed by an interlocking intramedullary nail in a 14 years old male osteosarcoma patient at distal femur. F. Intercalary allograft replacement fixed by an interlocking intramedullary nail in a 17 years old male osteosarcoma patient at the proximal tibia. G. Osteoarticular allograft replacement fixed by an interlocking intramedullary nail in 14 years old male osteosarcoma patient at distal femur. H. Osteoarticular allograft replacement fixed by a unilateral plate in 32 years old female osteosarcoma patient at proximal humerus.

The third step included bone implantation and fixation. The recycling bone (Fig. 1A–C), segment of fibular (Fig. 1B–D), or allografts (Fig. 1E–H) were placed in the bony defect and fixed by a unilateral plate (Fig. 1A,B,H) in 48 patients, by bilateral plates (Fig. 1C and D) in 20 patients and by intramedullary nailing (Fig. 1E–G) in 20 patients. Cancellous bone graft harvested from the iliac crest was applied at the osteotomy site in all cases. No fixation was applied in two patients (Fig. 3F) with the total humerus replacement (devitalized bone and allograft each).

### 2.5 Post-operation therapy and follow up

Only the osteosarcoma and Ewing sarcoma patients received other cycles of postoperative chemotherapy. All patients were examined every 3 months in the first 2 years, then 6 months in the next 2 years, and every year thereafter after surgery. The local plain X-ray, MRI or Doppler of involved limb and lung CT were taken at every examination for detection of local recurrence, pulmonary metastasis and to assess graft union.

The early proper exercise of the affected limb was performed in order to prevent muscle atrophy, joint stiffness, and deep vein thrombosis after surgery. Under the protection of braces, weight-bearing exercises were performed at 3 to 6 weeks after surgery. The involved joint movement started at 6 weeks after ligament repair in osteoarticular replacement patients. Weight-bearing movement and regular activities were allowed only after confirming the bone union.

### 2.6 Assessment of bone union and complications

The implanted bone union rate and time, complications including delayed union, nonunion, fracture, absorption, infection, and local recurrence were calculated.

Graft union was defined as an uninterrupted external bony border between the graft and the recipient bone in addition to obscured or absent osteotomy lines at both junctions according to radiograph assessment based on the radiographic union score system.^[24]^

### 2.7 Assessment of the oncological and functional effect

The functional status was determined at the final follow up using the Musculoskeletal Tumor Society Rating Scale (MSTS) score. This system was based on the analysis of 6 factors (pain, functional activities, emotional acceptance, use of supports for ambulation, walking ability, and gait). For each of the 6 factors, values of 0 to 5 were assigned based on established criteria. The result was expressed as a total with a maximum score of percentage.

The data were analyzed with SPSS software version 20.0. A *P* < .05 was considered statistically significant. Fisher exact test was used for categorical variables, whereas analysis of variance was used for continuous variables. Risk factors associated with the bone union and complications were performed by Kruskal–Wallis test in nonparametric tests for univariate analysis and binary logistic regression analysis for multivariate analysis. Survival rates were analyzed with the Kaplan–Meier method. The survival curve was drawn by Prism (version 8). Overall survival was taken from the date of diagnosis to the last date when the patient was documented to be alive or the date of death. Event-free survival was calculated from the time of histological diagnosis to the latest uneventful follow-up visit. An event was defined as a relapse or progression of the disease, a treatment-related secondary neoplasm, or death.

## 3 Results

### 3.1 Clinical and pathological outcomes

The operation time was 3 to 6 hours (4.62 hours on average), with the blood loss of 600 to 1500 ml (985.33 ml on average). The median length of the implanted bone segment was 18.57 cm (range, 5–38 cm). The implanted bone was entirely protected by internal fixation in 58 cases, incompletely in 20 cases, and with no fixation in 2 cases.

Sixty-seven patients had wide resection and 13 patients reached marginal resection. The surgical margins were clear with suspicious soft tissue and medullary cavity specimens at the osteotomy line by histopathological examination. Tumor necrosis rate was higher than 90% in 43, and less than 90% in 12 selected patients according to histological findings.

### 3.2 Functional results

Partial or full weight-bearing was permitted after a median of 7.61 months (range, 3–36 months). The Median MSTS score was 87.24% (range, 75%–100%) in intercalary graft patients, and 64.00% (range, 40%–100%) in osteoarticular graft patients at last follow-up (*P* = .001).

### 3.3 Implanted bone results

**Figure 2:**
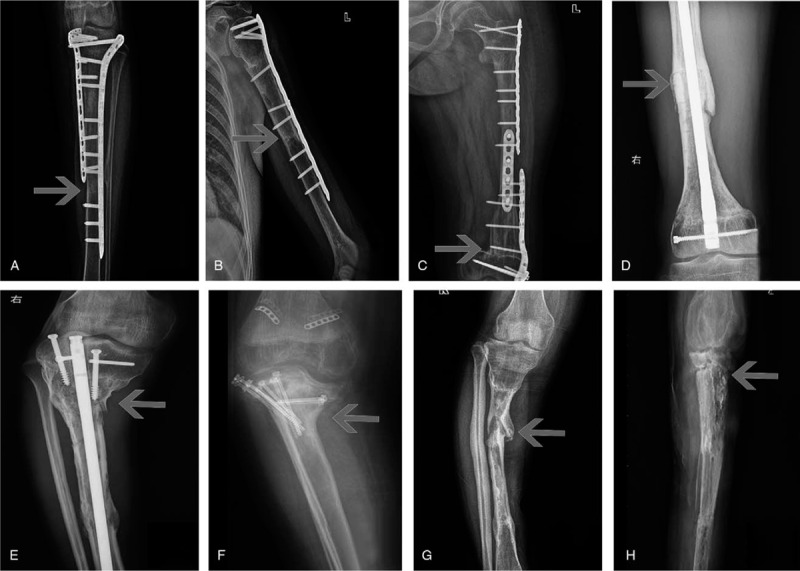
Union of implanted bone (the arrows show the involved bone). A. Primary union of an intercalary devitalized bone at 18 months after surgery in a 14 years old male osteosarcoma patient at the proximal tibia. B. Primary union of an intercalary allograft bone at 60 months after surgery in a 32 years old female chondrosarcoma patient at proximal humerus. C. Delayed union of an intercalary devitalized bone at 72 months after surgery in an 8 years old female osteosarcoma patient at distal femur. D. Primary healing of an intercalary allograft bone at 48 months after surgery in a 14 years old male osteosarcoma patient at distal femur. E. Malunion of an intercalary allograft bone at 120 months after surgery in a 17 years old male osteosarcoma patient at the proximal tibia. F. Malunion of an osteoarticular devitalized bone at 36 months after surgery in a 9 years old male osteosarcoma patient at the proximal tibia. G. Primary union with shift fracture of an intercalary devitalized bone at 120 months after surgery in a 14 years old female osteosarcoma patient after fixation has been taken out at proximal tibia. H. Bone union after infection and debridement with fixation taken out of an intercalary devitalized bone at 48 months after surgery in a 17 years old female osteosarcoma patient at the proximal tibia.

Complete bony union was achieved at the first stage in 23 out of 52 (44.23%) of intercalary graft patients at the metaphysis osteotomy sites with a median of 15.13 months (range, 4–31 months); in 26 out of 80 patients (32.5%) at the diaphysis sites with a median of 23.12 months (range, 6–60 months) (Fig. 2A,B,D). Bone healing time was different in metaphysis (14.58 ± 7.21 months) and diaphysis (24.47 ± 12.64 months) osteotomy sites (*P* = .002), devitalized autografts (14.50 ± 6.73 months) and allografts (34.09 ± 11.79 months) (*P* = .019). By univariate analysis, the factors related to bone healing included patient's gender (*P* = .023), age segment (*P* = .017), devitalization bone methods (*P* = .031, such as anhydrous alcohol, radiation, and liquid nitrogen allograft) and whether the implanted bone fixed completely (*P* = .000) (Table 1). By multivariate analysis, bone union was highly associated with devitalization bone methods (*P* = .01), implantation methods (*P* = .02) and tumor bone location (*P* = .05). Table 3 shows the estimated probability and 95% CI of bone union in one stage and the predictor variables (Table 3). Malunion occurred in seven cases including proximal tibia and distal humerus (Fig. 2E and F).

**Figure 3:**
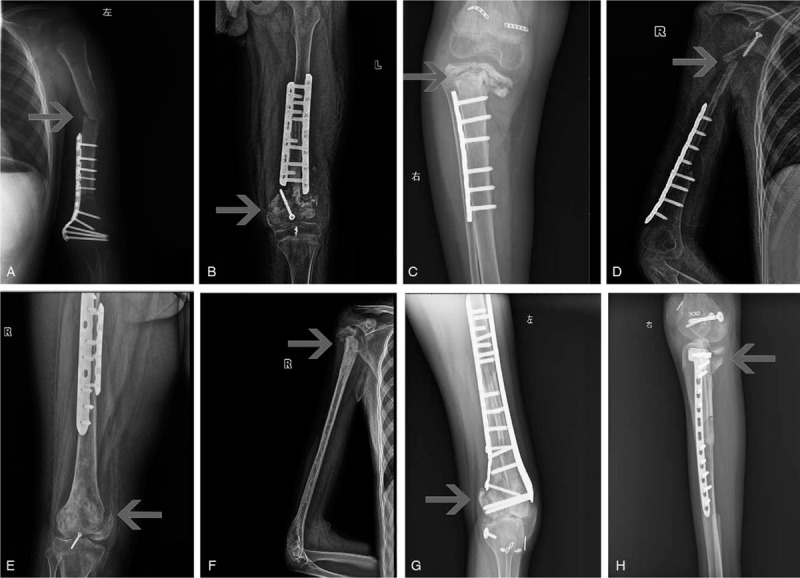
Implanted bone fracture in different biological reconstruction methods (the arrows show the fracture site). A. Shift fracture of osteoarticular allograft at 24 months after surgery in a 13 years old male osteosarcoma patient at proximal humerus. B. Metaphysis fracture and condyle collapse of osteoarticular devitalized bone at 18 months after surgery in an 11 years old female osteosarcoma patient at distal femur. C. Epiphysis slippage and condyle collapse of osteoarticular devitalized bone at 20 months after surgery in an 8 years old female osteosarcoma patient at the proximal tibia. D. Fibular segments fracture at 4 months after surgery in a 17 years old male osteosarcoma patient at proximal humerus. E. Condyle fracture of osteoarticular devitalized bone at 12 months after surgery in an 18 years old male osteosarcoma patient at distal femur. F. Metaphysis fracture and head absorption of total humerus devitalization at 28 months after surgery in a 23 years old female osteosarcoma patient. G. Condyle fracture of osteoarticular devitalized bone at 12 months after surgery in a 19 years old male osteosarcoma patient at distal femur. H. Condyle fracture of osteoarticular devitalized bone at 16 months after surgery in a 14 years old female osteosarcoma patient at the proximal tibia.

Delayed union was found in 9 (17.31%) of intercalary graft patients at the metaphysis osteotomy sites with a median of 15.33 months (range, 6–31 months), and in 6 (7.50%) patients at the diaphysis sites with a median of 30.33 months (range, 18–47 months). Delayed healing was achieved in 10 (12.50%) cases treated by revised surgery (Fig. 2C). The factors related to delayed union included devitalization bone methods (*P* = .011) and whether the implanted bone fixed completely (*P* = .043) by univariate analysis (Table 2). Single factor of devitalization bone methods (*P* = .02) was associated with delayed union by multivariate analysis (Table 3).

**Table 2:**
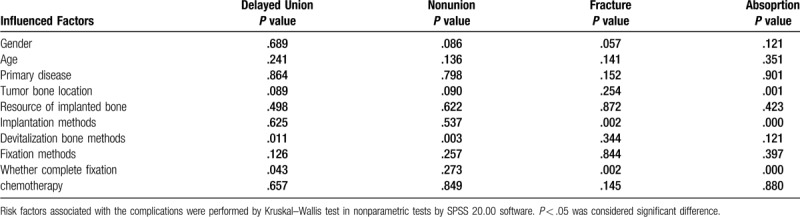
Risk factors related to complications.

**Table 3:**
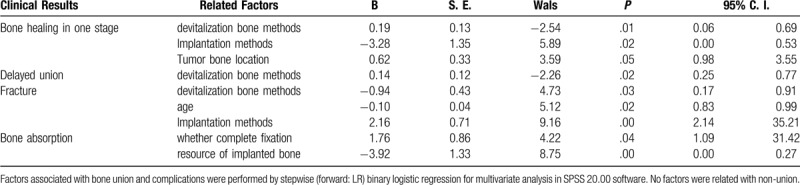
Factors influenced graft bone healing and complications.

**Figure 4:**
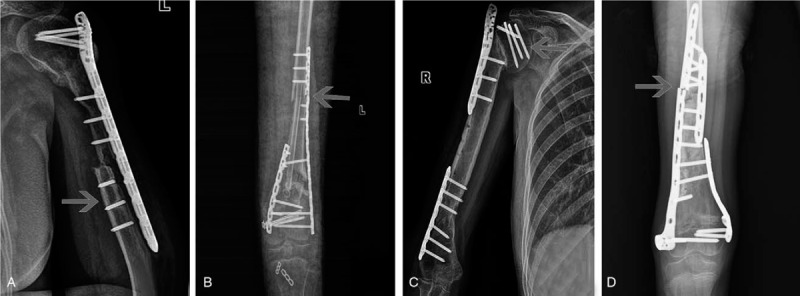
Fixation broken in different biological reconstruction methods (the arrows show the broken place). A. Broken screws with nonunion happened in an intercalary devitalized bone at 20 months after surgery in a 16 years old female osteosarcoma patient at proximal humerus. B. The broken plate was observed in an osteoarticular devitalized bone at 8 months after surgery in a 7 years old female osteosarcoma patient at distal femur. C. Broken screws with nonunion happened in an intercalary allograft bone at 36 months after surgery in a 21 years old female osteosarcoma patient at proximal humerus. D. The broken plate was observed in an intercalary devitalized bone at 15 months after surgery in an 8 years old female osteosarcoma patient at distal femur caused by trauma.

**Figure 5:**
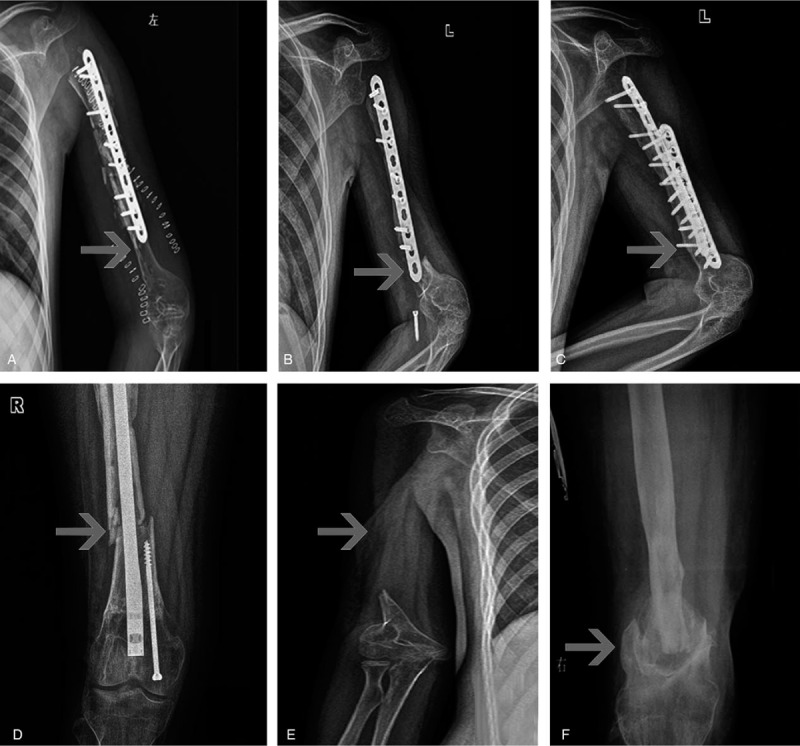
Long-term complications in implanted bone (the arrows show the involved bone or place). A. Fibular reimplantation with fixation change in a 14 years old male osteosarcoma patient at 48 months after the operation because of osteoarticular allograft bone resorption at proximal humerus. The bone union was seen at the graft junction. B. Re-fracture at the bone union junction with fixation loosen and broken was observed after 24 months of the first revised surgery. C. Bone union appeared at 12 months after the second revised surgery of autologous bone implantation and additional fixation. D. Fracture happened at 120 months after intercalary allograft bone operation at distal femur in a 15 years old female osteosarcoma patient. E. Complete implanted bone absorption in a 21 years old osteoarticular fibular replacement patient at 60 months after surgery at proximal humerus because of infection. F. Fracture and pseudo-articular formation in a 14 years old osteoarticular allograft replacement patient at distal femur at 168 months after the operation.

The fracture occurred in 25 (31.25%) cases, including 17 of devitalized bone graft, 4 allografts, and others. In univariate analysis, the factors were linked to fracture including implantation methods (*P* = .002) and whether graft bone complete fixation was achieved (*P* = .002) by univariate analysis (Table 2). Devitalization bone methods (*P* = .03), age (*P* = .02) and implantation methods (*P* = .00) were included factors by multivariate analysis (Table 3). The fracture appeared in 6 out of 11 patients (54.55%) younger than 10 years old, 14 of 47 patients (29.79%) between 10 and 20 years old. The fracture was observed in 9 (17.30%) cases with intercalary graft and 14 (50.00%) cases with osteoarticular graft. The fracture occurred in 12 (54.55%) cases with incomplete (Fig. 3A–F), and 11 (18.97%) cases with complete bone fixation (see Fig. 3G and H). Fracture accompanied by broken fixation appeared in nine cases (Fig. 4D). Delayed fracture after the fixation was taken out occurred in 5 cases (Figs. 2G, 5B and 5F), with the protection of the fixation in 3 cases (Fig. 5D). The fracture was treated by changing fixation with autologous bone implantation that was ultimately successful in 13 cases (Fig. 5C). The fracture was untreated in 5 cases (Fig. 3F). Fracture rate was 24.24% (8 of 33), 31.81% (7 of 22), and 44.44% (8 of 18) with different follow up periods of less than 24, 24 to 60, and more than 60 months (*P* = .146).

Nonunion happened in 22 (28.75%) cases, including 16 of devitalized bone (see Fig. 4A), 5 of allograft bone (Fig. 4C), and 1 case of fibular graft. Nonunion accompanied by broken fixation was observed in 13 cases. Factors related to nonunion were devitalization bone methods (*P* = .003) by univariate analysis. No factor included through multivariate analysis (Tables 2 and 3). Nonunion appeared in 9 (81.82%) cases with anhydrous alcohol devitalized bone, 2 (16.67%) cases with radiation bone, and 5 (27.28%) with liquid nitrogen bone. The nonunion rate was 21.43% (6 of 28), 58.33% (7 of 12), and 81.82% (9 of 11) in different follow up times of less than 24, 24 to 60, and more than 60 months (*P* = .01), respectively.

Absorption happened in 14 (17.50%) cases of implanted bone. The factors that influenced resorption were tumor bone location (*P* = .001), implantation methods (*P* = .000) and whether graft bone complete fixation achieved (*P* = .000) (Table 2) in univariate analysis. But resource of implanted bone (*P* = .00) and whether graft bone complete fixation (*P* = .04) were two related factors in multivariate analysis (Table 3). Absorption mainly happened in proximal humerus of 8 (57.14%) cases (Fig. 5A–C), and distal femur of 4 (10.81%) cases. Absorption appeared in 11 cases (39.29%) of the osteoarticular graft, and 3 cases (5.77%) of the intercalary graft (Fig. 5A and E). Absorption happened in 10 (45.45%) cases of incomplete (Fig. 5A), and 4 (6.90%) cases of complete graft bone fixation. The absorption rate was 9.10% (3 of 33), 22.73% (5 of 22), and 33.33% (6 of 18) in separate follow-up times of less than 24, 24 to 60, and more than 60 months (*P* = .037), respectively.

The infection was observed in 4 (5.00%) cases, including 2 cases of fibular and 1 case of devitalized bone and 1 case with allograft.

The revised surgery was performed in 28 (35%) cases, including 15 cases where fixation was changed with autologous bone implantation (Fig. 5A and C), 2 of bone implantation only, 8 cases with debridement and removed fixation (Fig. 2H), 2 cases with a changed prosthesis and 1 case of amputation. Finally, the implanted bone healed except for removing in 2 infection cases, and 1 in relapsed case.

### 3.4 Oncology results

**Figure 6:**
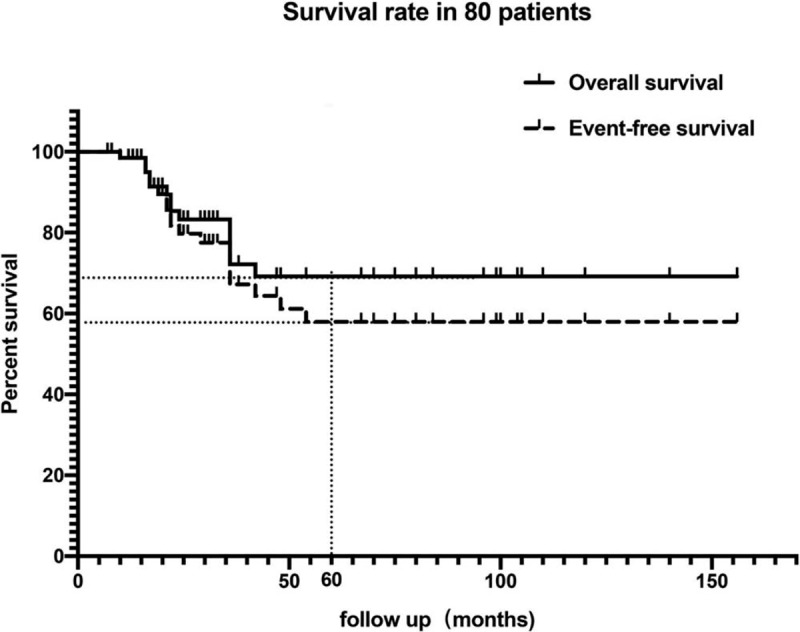
The event-free survival and overall survival curve in 80 patients.

The mean follow-up duration was 42.19 ± 36.43 months (range, 6–156 months), which was less than 24 months in 33 patients (40.7%), 24 to 60 months in 22 (27.2%) patients, and more than 60 months in 18 (22.2%) patients. At the end of the follow-up, there were 61 (76.3%) patients free of disease, 4 (5.0%) patients who survived with metastasis (including 2 of local recurrence and 2 of pulmonary metastasis), 15 (18.8%) patients who died. At 5 years, event-free survival was 58%, and overall survival was 69% (Fig. 6). Local recurrence was dealt with tumor resection and prosthesis replacement in one and amputation in another patient. Two patients with pulmonary metastasis were treated by chemotherapy.

## 4 Discussion

The most commonly used biological reconstructions include allograft, autologous bone. In this study, we found that the healing time is shorter in devitalized autografts than in allografts, in metaphysis than in diaphysis osteotomy sites. The devitalization methods include

(1) irradiation,
(2) autoclaving,
(3) pasteurization, and
(4) freezing-thawing with liquid nitrogen.

Because autoclaving or pasteurization are limited by the thermally-induced weakness of bone and loss of osteoinductive properties,^[18]^ we mainly used anhydrous alcohol, liquid nitrogen, and radiation due to low tumor recurrence with no difference in these methods. Yet, the nonunion rate was higher in anhydrous alcohol devitalized method (81.82%) than in other methods, partly because of the extended follow-up period and low ability to revascularization and cell repopulation from the surrounding soft tissue.

We found more improved joint movement, higher MSTS score, higher union rate, lower fracture rate, and lower graft bone absorption rate in intercalary graft than in osteoarticular graft patients, which indicates that biological reconstructions are mainly suitable for the massive intercalary bone defect. Joint instability, degeneration, and stiffness are the side effects that influence the function of the involved joint after osteoarticular replacement.

We also found a different pattern of bone healing in fixation methods of the intramedullary nail and extramedullary plates. Graft fixed by intramedullary nail had a large amount of external callus formation with a visible fracture line several years after surgery (Fig. 2D). The external callus formation was not seen in patients with an extramedullary plate fixed. Moreover, the fracture line also disappeared early (Fig. 2A–C) in these patients. More interestingly, micro-movement was observed at the surface of the implant-host bone in some revision surgeries because of nonunion after intramedullary needle fixation, which may be the reason for the formation of external callus. Therefore, in order to reach the rapid graft bone healing, bilateral locked plates or intramedullary needle combined plates were recommended as the first choice.

The complication rates in biological reconstruction include (31.25%) for fracture and (28.75%) for nonunion, which is relatively high. Because the re-implant bone lacks blood supply and nutrition, it can only function as a frame structure without the capability to respond to changing biomechanical demands. Integration of the graft is exclusively driven by the recipient bed, which is often weakened by chemotherapy, radiation, and a lack of soft tissue coverage due to tumor resection.^[25]^ Consequently, nonunion and fractures quickly appear. The implanted bone healing is mainly the process of “creeping substitution,” whereby osteoinduction, osteoconduction, and neoangiogenesis gradually advance at the bony junction. In other studies, postoperatively evaluated biopsy specimens harvested from pasteurized bone for more than 3 years, where the cortices of the graft remained necrotic, with empty osseous lacunae. We found fracture at the shift and no new bone formation in the implanted cortical bone 120 months after operation in one patient (Fig. 5D). The fracture also happened at the bony junction after the fixation was taken out in one patient (Fig. 5B). These phenomena illustrate that the repopulation of implanted bone was very slow, especially in the superficial layer of cortical bone. Although the union was achieved after a reasonable time, partial substitution of the whole graft takes years, and a transformation into physiological bone remains incomplete. Due to the slow integration and its initial biomechanical properties, fractures can happen the whole period of follow up from 6 months to 60 months with a rate of 24% to 45%. The nonunion rate increased with the prolonged follow-up time, which was 21.43% in less than 24 months with an increase to 81.82% in more than 60 months. No influence of chemotherapy on bone nonunion or absorption was observed in this study.

The unique complication in biological reconstruction is re-implanted bone absorption, which occurred at the higher rate that occurred in the proximal humerus (57.14%), osteoarticular graft with incomplete internal fixation, especially with the unilateral plate. The absorption place included a humerus head and an un-fixed implanted bone shift (Figs. 3A and 5C). Besides the inadequate soft tissue coverage and little blood supply, insufficient fixation with continuous shear stress on the cortical bone can lead to microfracture. Continuous movement without fixation results in a more extensive range of fractures and bone resorption without remodeling. The absorption rate also increased with prolonged follow-up time without intervention.

In this study, we observed the close relationship between fracture, delayed union, absorption with whether completely fixed the implanted bone. Without the protection of a robust internal fixation, the transplanted bone can be easily fractured and resorbed during the postoperative limb movements. Even if a bone union is achieved, removing the internal fixation can lead to the re-fracture of the grafted bone. Due to the long period of the healing process of implanted bone, which is different from the regular bone union, it is recommended to use continued and stable internal fixations such as bilateral cortical plates, which prolong the period of normal movement of the involved limb.

The limitations of this study are the retrospective character, a small number of cases, incomplete clinical and follow-up data, sole focus on the location of femur, tibia, and humerus. A prospective study with more patients and improved surgical techniques should be performed in the future.

## 5 Conclusions

Even though biological reconstruction is accompanied by various complications such as fracture, nonunion and absorption of massive implanted bone, it is still beneficial for preserving the anatomical structure and normal joint function of the involved extremity, especially with reference to long-term effect in young patients with sarcoma occurring in the most common sites of femur, tibia, and humerus with intercalary reconstruction. Persistent and robust internal fixations with sufficient blood supply and soft tissue coverage are the main factors that can improve implanted bone healing and reduce postoperative complications.

## Author contributions

All authors read and approved the final manuscript.
